# Evaluation of Culture Media for Isolation of Mycobacterium Species from Human Clinical Specimens

**DOI:** 10.7759/cureus.757

**Published:** 2016-08-30

**Authors:** Padmavali Palange, Rahul Narang, Venkataramana Kandi

**Affiliations:** 1 Department of Microbiology, Prathima Institute of Medical Sciences; 2 Department of Microbiology, Mahatma Gandhi institute of Medical Sciences, Sevagram, Maharashtra.

**Keywords:** blood agar, mycobacteria, pulmonary, extrapulmonary

## Abstract

Background: Laboratory diagnosis of tuberculosis has undergone a rapid change during last few years and a number of techniques for culture as well as molecular diagnosis have been used with their respective advantages and disadvantages. Sporadic studies have also reported the isolation of *M. tuberculosis *on standard blood agar (BA), which at one time was not considered as a suitable medium for mycobacterial culture. The present study was conducted to evaluate the routine use of 5% sheep BA in a mycobacteriology laboratory by comparing isolation rates and time for isolation of mycobacteria from pulmonary and extrapulmonary samples with those on Lowenstein-Jensen (LJ) medium.

Material and Methods: BA with antibiotics was prepared and dispensed as slants in McCartney bottles. LJ was prepared and dispensed following the Revised National Tuberculosis Control Programme (RNTCP) guidelines. A total of 500 suspected tuberculosis samples were inoculated on both media in duplicate, incubated at 37^0^C, and observed daily until the appearance of growth.

Results: Out of 500 inoculated samples, 99 showed growth on BA and 112 showed growth on LJ medium. Mean growth time on BA was less as compared to LJ medium. The contamination rate was found to be more on BA (7.2%) than on LJ (4.8%).

Conclusions:  Mycobacterial growth time was less on BA as compared to LJ.

## Introduction

In recent years, tuberculosis management has changed significantly. Considering the impact of multidrug-resistant tuberculosis (MDR-TB), the Programmatic Management of Drug Resistant TB (PMDT) services have been implemented in almost all the high MDR-TB burden countries, including India. During last 10 years, the World Health Organization (WHO) has approved various rapid techniques for the isolation and identification of *M. tuberculosis* that include the mycobacterial growth indicator tube (MGIT), lateral flow assay, and drug susceptibility testing (DST) using line probe assay and GeneXpert (Cepheid, Inc., Sunnyvale, CA) [1]. These newer technologies have been implemented in selected laboratories under the Expanding Access to New Diagnostics for Tuberculosis (EXPANDx) TB Project. However, these techniques are expensive and technically demanding, and it may not be possible for the non-funded laboratories to implement them. Thus, there is a need for other rapid systems which are financially and technically not that demanding.

The BACTEC™ MGIT™ 960 automated system (Becton, Dickinson & Co., Franklin Lakes, NJ) has been routinely used for liquid culture and drug susceptibility testing. Molecular tests are not available at all places. During the renaissance of TB diagnostics, culture for mycobacteria has been pushed to the back seat [2].

In fact, the culture for *M. tuberculosis* provides a definitive diagnosis of tuberculosis by establishing the viability and identity of the organisms [1]. Even under the programme conditions, cultures increases the number of TB cases found, often by 30-50%, and detects cases which are smear-negative. Since culture techniques detect fewer bacilli, the efficiency of diagnosing cases of failure at the end of treatment can also be improved considerably. Cultures also provide sufficient material for drug susceptibility and identification tests [1, 3-4]. Agar-based and egg-based solid media incorporating malachite green have been recommended as the “gold standard” for the isolation and definitive diagnosis of *M. tuberculosis* for quite some time [5].

In recent times, more attention has been devoted to liquid media that are more attractive as they offer significantly shorter turnaround times for the detection of mycobacteria. Nucleic acid amplification diagnostic technologies have also been promoted, owing to their rapidity, sensitivity, and specificity [6].

However, these newer technologies are expensive, and they require sophisticated machines, laboratories, and a high degree of technical expertise. Such requirements pose logistic and economic problems, especially in resource-limited areas where bacteriological culture facilities are few and the prevalence of tuberculosis is high [7].^ ^The available laboratories, which have financial constraints, require culture media or techniques that are rapid and inexpensive.

Blood agar (BA) was used to isolate the *Mycobacterium* species in the 1960's by a few researchers [8-11]. However, after that, it was forgotten until reutilization by Drancourt, et al. who first reported the incidental growth of *M. tuberculosis *colonies on blood agar and termed this finding the “end of dogma” [5]. This medium has the potential of replacing egg- and agar-based media and is cost-effective, more sensitive, and rapid.

Therefore, the present study was aimed to evaluate the routine use of 5% sheep BA in a TB laboratory by comparing isolation rates and time for isolation of mycobacteria using morphology and biochemical tests from pulmonary and extrapulmonary samples with those on Lowenstein Jensen's (LJ) medium.

## Materials and methods

This hospital-based prospective study was conducted in the department of microbiology, rural hospital of central India from March 2011 to July 2012. A total of 500 samples received in mycobacteriology laboratory from patients attending a rural hospital which included 200 pulmonary and 300 extrapulmonary samples. The study was approved by Institutional Ethics Committee. Informed patient consent was obtained.

Out of 200 pulmonary samples, 98% were sputum and 2% were bronchoalveolar lavage (BAL). Among 300 extrapulmonary samples included were sterile body fluids (67.4%), pus (16.3%), endometrial tissues (13%), and urine (3.3%).

### Preparation of culture media

Sheep blood was collected from the jugular vein of sheep with 50 ml syringe in a sterile bottle containing sodium citrate (1:9) as an anticoagulating agent and stored at 2-8° C until use. For preparing BA, sodium chloride (0.5 g), peptone (1 g), meat extract powder (1 g), and agar (2 g) was added to 100 ml of distilled water, and pH was adjusted to 7.2-7.4. This mixture was sterilized by autoclaving at 121° C for 15 minutes. After cooling to 45-50° C, 5 ml of citrated sheep blood was mixed in it. To avoid growth of contaminants, 90 µl of nystatin solution (prepared by dissolving 18 mg nystatin dissolved in 9 ml methanol) and 50 µl of antibiotic solution (prepared by dissolving 88.8 mg polymixin-B, 5 mg trimethoprim, and 20 mg nalidixic acid in 5 ml distilled water) were added to the media. Contents were mixed well and approximately 12-13 ml was quickly dispensed into 30 ml flat bottom culture tubes (McCartney bottles). These bottles were kept obliquely and allowed to cool till slants of blood agar were ready. Instead of using Petri plates, we used McCartney bottles for blood agar to avoid desiccation. To check sterility, these bottles were incubated at 37° C for 24 hours and the set of bottles showing any growth were discarded. Remaining media were then stored at 2-8° C for further use. LJ medium was prepared as per the recommendations of RNTCP [3].^ ^*M. tuberculosis* H37Rv and *M. fortuitum *were inoculated on LJ as positive controls for *M. tuberculosis* and non-tuberculous *Mycobacterium* species (NTM), respectively.

Pulmonary and other potentially contaminated extrapulmonary samples were processed by modified Petroff’s method. Other extrapulmonary samples from sterile sites were directly inoculated. A set of BA and LJ media were inoculated with 50 µl sample. They were incubated at 37° C and observed daily until the appearance of growth. Smears were prepared from isolated colonies and identified by Ziehl-Neelsen's staining method. 

Paired t-test was used to compare the number of days taken for the appearance of visible growth on BA and LJ medium. After microscopic examination, all the isolates were identified as *M. tuberculosis *or NTM by performing biochemical tests (niacin production, nitrate reduction, heat stable catalase, and susceptibility to p-Nitrobenzoic acid medium) recommended by RNTCP's standard operating procedures (SOP) [3].

## Results

Out of 500 samples processed for Ziehl-Neelsen’s (ZN) staining, 100 (50%) pulmonary samples and 26 (9%) extrapulmonary samples were smear-positive.

Culture on BA and LJ medium revealed growth in 99 and 112 samples, respectively. The detailed report of smear and culture positivity is shown in Table 1.

**Table 1:** Growth of Pulmonary and Extrapulmonary Samples on BA and LJ with Respect to Smear Status

Total Samples (500)	Smear Status	BA (%)	LJ (%)
Pulmonary (200)	Positive (100)	75 (75)	84 (84)
	Negative (100)	8 (8)	8 (8)
Extrapulmonary (300)	Positive (26)	10 (38.5)	13 (50)
	Negative (274)	6 (2.2)	7 (2.5)

All the samples showing contamination either on BA or LJ or both media were excluded from the study.

**Figure 1:**
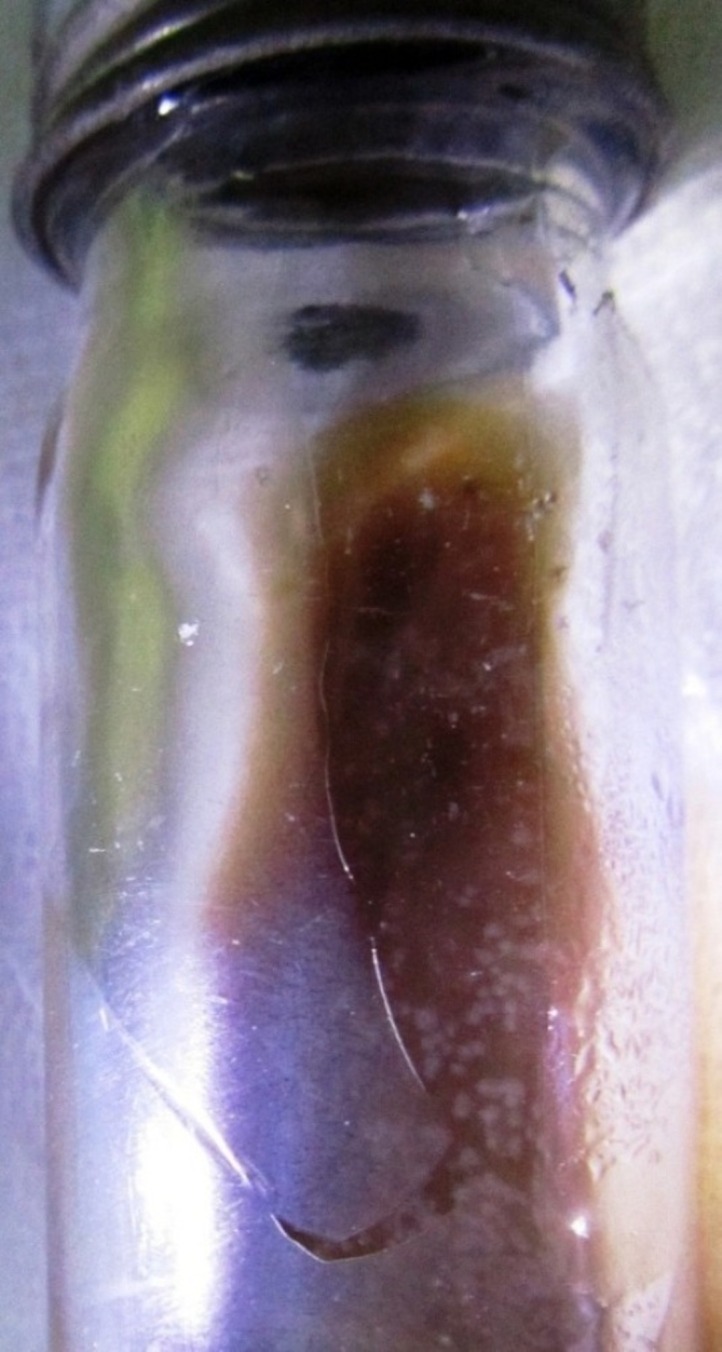
Blood agar (BA) medium showing growth of Mycobacterium tuberculosis.

On BA,  small, light grayish, glistening, convex, dry, irregular, easily recognizable colonies against a red background were observed. They were not easily emulsifiable and were later identified as *M. tuberculosis *as shown in* *Figure 1.

**Figure 2:**
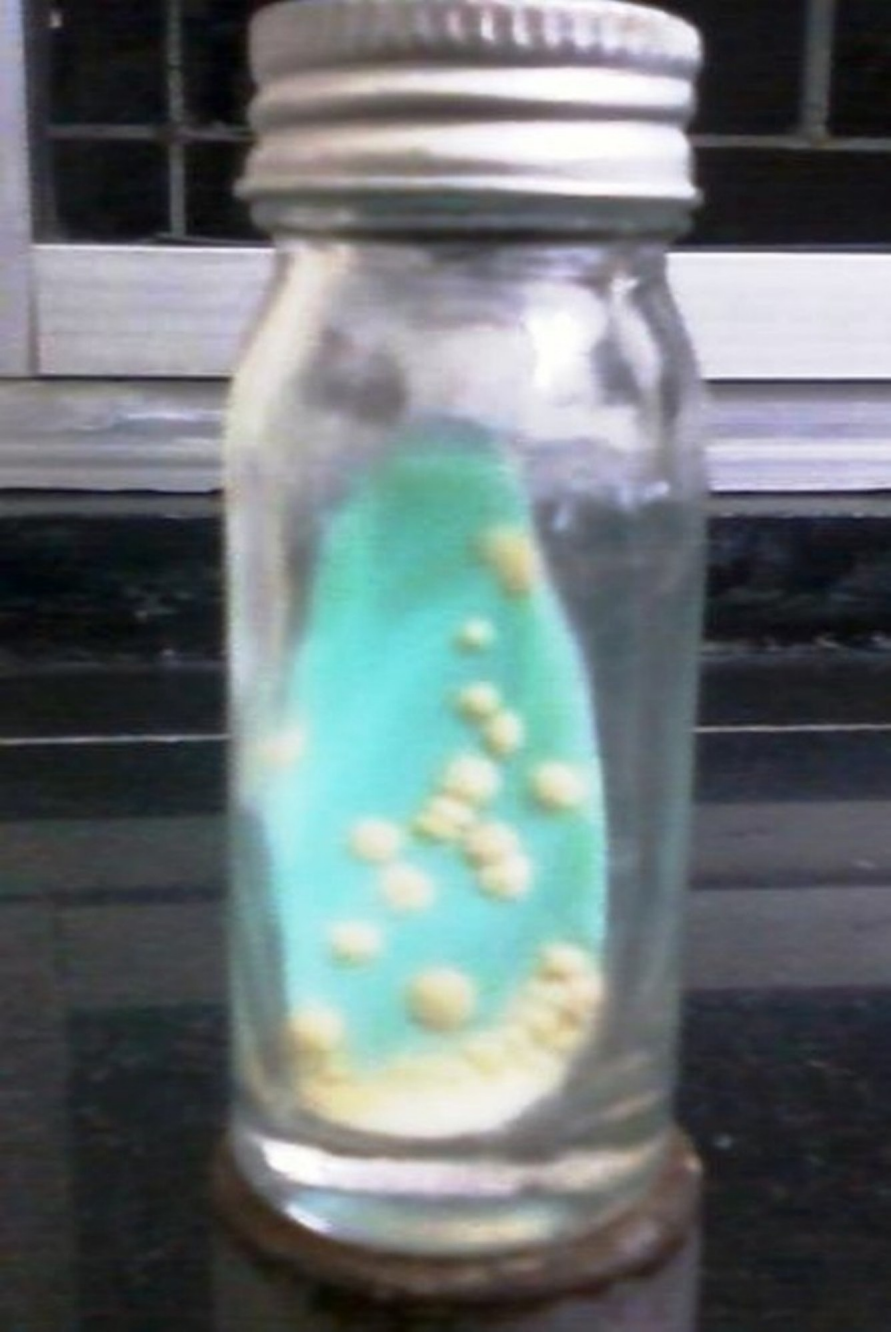
Lowenstein Jensen's (LJ) medium showing growth of Mycobacterium tuberculosis.

Colonies of *M. tuberculosis* on LJ medium were cream colored, dry, rough, raised, irregular with a wrinkled surface, and not easily emulsifiable as shown in Figure 2.

Colonies, which were flat, smooth, moist, white, easily emulsifiable, and growing rapidly either with or without pigment production, were identified as NTM.           

Mean growth time for both pulmonary and extrapulmonary samples was less on BA as compared to LJ medium, and this difference was statistically significant as shown in Table 2.

**Table 2:** Median Growth Time for Pulmonary and Extrapulmonary Samples

Samples	Growth Time on BA	Growth Time on LJ
Pulmonary	18.4 days	31.32 days
	(Range: 18.37 - 18.44 days)	(Range: 30.8 – 31.64 days)
Extrapulmonary	15.7 days	40.67 days
	(Range: 15.7 - 15.38 days)	(Range: 39.25 – 41.86 days)

The culture contamination rate was found to be more on BA (7.2%) than on LJ medium (4.8%).

All the isolates were identified as* M. tuberculosis* and NTM by standard laboratory identification methods. Four isolates grown on BA and LJ medium were identified as NTM. Ninety-five isolates on BA and 108 isolates on LJ medium were identified as *M. tuberculosis*.

## Discussion

Recently, there has been a lot of interest in the use of blood agar for isolation and DST of *M. tuberculosis*. The studies conducted previously by Drancourt, et al., Satti, et al., and Mekasha have positively evaluated the utility of BA for primary isolation as well as DST of *M. tuberculosis* [7, 12-13]. An Indian study reported by Mathur, et al. also used BA for primary isolation of *M. tuberculosis* from pulmonary samples [14].

In the present study, we have used blood agar to isolate *M. tuberculosis* from both pulmonary as well as extrapulmonary samples. The culture sensitivity of BA was 89.3% for pulmonary isolates, which was lower than those reported by Drancourt, et al. (98.9%), Satti, et al. (90.9%), Mekasha (98%), and Mathur, et al. (94.2%) [7, 12-14]. This can be explained by the fact that we had used RNTCP-advocated modified Petroff’s method (4% NaOH) for sample processing while, in others studies, the NALC-NaOH method was used [3]. The higher alkalinity of 4% NaOH might not have been neutralized by distilled water and could have led to reduced growth on BA. LJ medium, on the other hand, has sufficient buffering capacity and can neutralize the high alkalinity and can improve the culture sensitivity [15]. 

Among 100 smear-positive pulmonary samples, only 84% showed growth on the LJ medium, which was lower than previous reports by Tarshis, et al. (89.9%), Tarshis, et al. (90.1%), Satti, et al. (95.7%), and Mathur, et al. (97.1%) [8-9, 12, 14]. Only 75% smear-positive cases revealed growth on BA, which was lower than that reported by Tarshis, et al. (93.6%), Tarshis, et al. (94.2%), Maccabe, et al. (78.26%), Dunlop, et al. (87.5%), Satti, et al. (97.9%) [12], and Mathur, et al. (94.2%) [8-12, 14]. Low isolation rates in our study could be attributed to sample decontamination with 4% NaOH or prior anti-tubercular treatment [16].

In our study, the specificity, positive predictive value, and negative predictive value of culture on BA was observed to be 99.59%, 93.75%, and 98.02%, respectively. These values were in agreement with those obtained in a study by Mekasha from Ethiopia [13].

Among the smear-positive extrapulmonary samples, 50% showed growth on LJ medium and only 38.5% revealed growth on BA. Among the smear-negative samples, the positivity on LJ medium and BA was 2.5% and 2.2%, respectively. For extrapulmonary samples, BA sensitivity was 38.5% as compared to LJ medium at 50%. No other previous study has reported the sensitivity and specificity for extrapulmonary samples. We had specifically included a higher number of extrapulmonary samples (n = 300) as we wanted to study the utility of BA for primary isolation. A laboratory in France has started using BA for primary isolation of *M. tuberculosis* from pulmonary and extrapulmonary samples [5].

Results of the present study showed a significant difference (p-value < 0.0001) in time to detect *M. tuberculosis *culture positivity on BA and LJ medium. Isolation time was less on BA as compared to LJ medium. The time for culture positivity of pulmonary samples on BA ranged from three to 35 days with median time to detection being 18.4 days (16.4 - 18.9 days, 95% CI). On the other hand, culture positivity in LJ media ranged from three to 63 days with median time to detection being 31.32 days (27.7 - 32.7 days, 95% CI). The study results were in agreement with Tarshis, et al. who reported time for culture positivity on BA (18.9 days) and LJ medium (21.5 days) [8]. A study by Maccabe, et al. also revealed similar observations. They included 36 microscopically positive sputum samples and growth was noted within 14 days on saponated BA [10]. Drancourt, et al. compared the time for growth of 38 pulmonary and extrapulmonary samples and found that 21 had grown on BA after six days of incubation, and all the samples were culture-positive within two weeks [5]. The same study has reported a median time for isolation as 19 ± 5 days using BA. Mathur, et al. observed that the mean time to detect *M. tuberculosis* on BA was 13.6 ± 5.2 days as compared to 20.4 ± 5.1 days on LJ medium [14]. Mekasha observed the time for culture positivity on BA media ranged from seven to 32 days with a mean time of 17.3 days (15.8 - 18.7 days, 95% CI) [13]. On the other hand, culture positivity on LJ media ranged from 10-41 days with a mean time of 22.7 days (20.8 - 24.6 days, 95% CI). Satti, et al. reported time to detect growth on BA as seven days [12]. Our findings were in contrast to those reported by Dunlop, et al. who observed average growth time as 10 days on BA and 9.3 days on LJ medium [11].

Colony characters on BA observed in our study were small, rough, grayish colored, and glistening against a red background. The size of the colony increased to a certain extent but then remained the same size later. A similar description of the colonies has been given by other researchers who have worked on BA for *M. tuberculosis* isolation. Tarshis, et al. reported that on initial isolation the colonies were pinpoint in size, light gray, glistening, and easily recognized against the red background of the blood when examined under a bright light. They gradually increased in size and reached maximum growth in about five to six weeks, where mature colonies varied greatly in diameter, measuring approximately 1 to 10 mm, and assumed a tannish gray or red color. It was also noted that some colonies became dark tan with a slight greenish cast [8-9]. In their study, Maccabe, et al. noted early growth on BA showing small, circular, bluish-grey, and fairly moist convex colonies having an entire edge. After about three weeks, the center of the colony changed to brown, eventually involving the entire colony. It was also observed that the colony started to become dry and irregular after six weeks. After two months, some colonies exhibited a depressed center. At this stage, the colony was about 2 mm in diameter [10]. Mathur, et al. and Satti, et al. described colonies of *M. tuberculosis* on BA as light grayish, glistening, and easily recognized against a red background of BA as compared to rough, cream-colored colonies on LJ medium against bluish-green backdrop [12, 14]. 

In our study, the contamination rate was found to be high in BA (7.2%) than in LJ medium (4.8%). In contrast to the results of our study, some studies have reported lower contamination rates on BA compared to LJ medium. Tarshis, et al. reported 5% contamination for BA medium and 6.9% for LJ medium [8]. In the other report from the same author, 5% contamination was noted on BA and 7.1% for LJ medium [9]. Mathur, et al. observed a contamination rate of 1.6% on BA and 7.8% on LJ medium [14]. In their study, Satti, et al. reported contamination rates of 2.8% and 4.2% on BA and LJ medium, respectively [12].

In our study, the cost to prepare BA was 8.60 Rs/- ($0.13) per sample and the cost of LJ medium came to be 6.20 Rs/- ($0.09) per sample. The total cost for culturing one sample on BA was 22.02 Rs/- ($0.33), and on LJ medium, it was 16.02 Rs/- ($0.24).

## Conclusions

Thus, it can be concluded from this study that BA is a rapid growth medium for the primary isolation of *M. tuberculosis* from both pulmonary and extrapulmonary samples. It can be used as a culture media for routine isolation of mycobacteria in resource-limited settings. However, due to increased contamination rate on BA, laboratories should follow improved procedures for specimen decontamination. Although the cost of culture on LJ medium is lower as compared to BA, preparation of LJ medium requires skilled technical personnel and additional laboratory infrastructure. We feel that further studies should be undertaken to assess BA as growth media for mycobacteria.
